# Expression of PD-L1, TIGIT, and CD155, and Human Papillomavirus Status in Patients with Advanced Penile Cancer

**DOI:** 10.1016/j.euros.2025.07.012

**Published:** 2025-08-09

**Authors:** Emma Ulvskog, Peter Kirrander, Erik K. Persson, Gabriella Lillsunde-Larsson, Luiza Dorofte, Tania Franceschini, Michelangelo Fiorentino, Sabina Davidsson

**Affiliations:** a Department of Oncology, Faculty of Medicine and Health, Örebro University, Örebro, Sweden; b Department of Urology, Faculty of Medicine and Health, Örebro University, Örebro, Sweden; c Regional Cancer Centre Mid Sweden, Uppsala, Sweden; d Department of Laboratory Medicine, Faculty of Medicine and Health, Örebro University, Örebro, Sweden; e School of Health Sciences, Örebro University, Örebro, Sweden; f Pathology Service Maggiore Hospital, Bologna, Italy; g Department of Medical and Surgical Sciences, Alma Mater University of Bologna, School of Medicine, Bologna, Italy

**Keywords:** Penile cancer, Chemotherapy, Radiotherapy, Tumor microenvironment, TIGIT, PD-L1, CD155, Human papillomavirus

## Abstract

**Background and objective:**

To improve treatment for patients with penile cancer, there is a need for prognostic and treatment predictive biomarkers. The objective of this study was to examine the expression of checkpoint proteins (programmed cell death ligand 1 [PD-L1], T-cell immunoglobulin and immunoreceptor tyrosine-based inhibitory motif domain [TIGIT], and cluster of differentiation 155 [CD155]) and human papillomavirus (HPV) status in primary tumors of penile cancer patients with indication for perioperative oncological treatment. As a secondary aim, we evaluated the associations between these biomarkers and penile cancer–specific survival.

**Methods:**

Fifty-two patients who underwent surgical treatment during 2009–2018 were included. HPV status was determined by polymerase chain reaction, and tissue microarray sections from primary tumors were subjected to immunohistochemistry to evaluate the expression of PD-L1, TIGIT, and CD155.

**Key findings and limitations:**

PD-L1, TIGIT, and CD155 were expressed widely. Specifically, 75% of patients had PD-L1–positive tumors, 80% had TIGIT-positive tumors, and 98% had CD155-positive tumors. Additionally, 47% of patients had HPV-positive tumors. Patients with HPV-positive tumors had better survival than those with HPV-negative tumors. Patients with indication for perioperative oncological therapy who received such treatment and whose tumors exhibited low PD-L1 expression demonstrated better survival than those with higher PD-L1 expression levels. The main limitations of this study include the small number of patients, retrospective design, and use of tissue microarrays rather than whole tissue sections.

**Conclusions and clinical implications:**

We found high expressions of the investigated checkpoint proteins, suggesting an immunosuppressed tumor microenvironment in patients with advanced penile cancer. These findings imply that checkpoint proteins could serve as prognostic and treatment predictive biomarkers. Patients with HPV-positive tumors had better prognosis.

**Patient summary:**

In this report, we investigated tumor tissue from patients with penile cancer and identified a high prevalence of proteins that play an important role in the immune system’s defense against cancer. The findings suggest that these proteins can be important in understanding and developing treatments for patients with lymph node metastasized penile cancer.

## Take Home Message

We report on high expression of immune checkpoint proteins PD-L1, TIGIT, and CD155 in men with lymph node metastasized penile cancer. We also found that human papillomavirus positivity was correlated with better survival.

## 1 Introduction

Penile cancer is a rare form of squamous cell carcinoma known to arise from either of two etiological pathways: one related to chronic inflammatory conditions and the other related to human papillomavirus (HPV) infection [1]. Lymph node involvement is the strongest predictor of survival [2,3], and international and national guidelines recommend perioperative oncological treatment to improve prognosis for patients presenting with lymph node metastases [4]. However, the evidence supporting recommendations is weak, and currently, there are no biomarkers to guide treatment decisions. HPV is a well-known risk factor as well as a possible positive prognostic marker for penile cancer. It has also been suggested to be a predictive biomarker for treatment response in various squamous cell carcinomas [[5], [6], [7]].

Other potential biomarkers are proteins related to the immune response to cancer. Immune checkpoint proteins are examples of such proteins, and checkpoint inhibitors have become established treatment options for several types of squamous cell carcinomas. Currently, the primary targets are the immune checkpoint protein programmed cell death protein 1 (PD-1) and its ligand (PD-L1). PD-1/PD-L1 expression is present in several malignancies and holds prognostic value, also for penile cancer [[8], [9], [10]]. Treatment with checkpoint inhibitors has been shown to be effective only in a smaller proportion of penile cancer patients [[11], [12], [13]].

Beyond PD-1 and PD-L1, there are many checkpoint proteins that could prove to be biomarkers in the future. In this study, we investigate the immune checkpoint proteins T-cell immunoglobulin and immunoreceptor tyrosine-based inhibitory motif domain (TIGIT) and its ligand (cluster of differentiation 155 [CD155]). TIGIT and CD155 have been suggested as promising new targets for cancer immunotherapy [14,15]. Interaction between TIGIT and CD155 leads to the inhibition of T-cell activation and facilitates immune escape. TIGIT and CD155 expression has been proposed as a marker of prognosis in several cancers [16], and in 2021, the U.S. Food and Drug Administration approved the first commercially available anti-TIGIT antibody tiragolumab.

Identification of prognostic and predictive biomarkers for penile cancer would be highly beneficial, in both the perioperative and the palliative setting. Therefore, the primary aim of this study was to examine the expression of PD-L1, TIGIT, and CD155, and the HPV status in the primary tumors from penile cancer patients with indication for perioperative oncological treatment according to the present Swedish national guidelines. As a secondary aim, we evaluated the association between these biomarkers and penile cancer–specific survival (peCSS).

## 2 Patients and methods

### 2.1 Patients

Fifty-two consecutive penile cancer patients who underwent surgical treatment at Örebro University Hospital between 2009 and 2018 were included in this study. All patients had at least two unilateral lymph node metastases without evidence of distant metastases, thereby meeting the criteria for perioperative oncological treatment according to the Swedish national guidelines. Information on oncological treatment was extracted from a previously constructed database [2,17].

This study was approved by the Swedish Ethical Review Authority.

### 2.2 Immunohistochemistry analysis of PD-L1, TIGIT, and CD155

Tissue microarray (TMA) sections (4 µm) retrieved from previously constructed TMAs, comprising four tissue cores (0.6 mm) from the primary tumor, were subjected to immunohistochemistry. TMAs were taken from different regions of the invasive component of the tumor, chosen on microscopical assessment. To assess heterogeneity, tumor regions with different morphologies were chosen if these were not necrotic. A pathologist (L.D.) marked the areas with viable tumor cells, without necrosis, from the middle of the tumor as well as from the invasive tumor front.

TMA tissue sections underwent deparaffinization and staining with an anti–PD-L1 antibody (VENTANA, PD-L1 clone SP263, rabbit monoclonal, prediluted; Ventana Medical Systems, Tucson, AZ, USA) using the Benchmark XT staining system. Detection was carried out using the OptiView Universal DAB Detection Kit (Ventana Medical Systems). Additionally, staining was performed using an anti-TIGIT antibody (TIGIT clone BLR047F, rabbit monoclonal; BioCare, Pacheco, CA, USA) and an anti-CD155 antibody (Anti-poliovirus receptor/PVR ab230338, rabbit polyclonal; Abcam, Cambridge, UK) on the IntelliPATH staining system, with detection conducted using the HRP-Polymer Detection kit. Subsequently, all sections were counterstained with hematoxylin, dehydrated, and mounted. Tonsil tissue served as the positive control for TIGIT, while breast cancer tissue was used for CD155.

### 2.3 Evaluation of PD-L1, TIGIT, and CD155 expression

#### 2.3.1 PD-L1

The staining was assessed independently by two experienced uropathologists (T.F. and M.F.) blinded to the patients’ clinicopathological characteristics. Since there are no guidelines for PD-L1 evaluation for penile squamous cell carcinoma, the evaluation was performed according to the *PD-L1 IHC 22C3 pharmDx—head and neck squamous cell carcinoma (HNSCC)* interpretation manual [18,19].

For PD-L1 expression, we used the combined positive score (CPS).

Any partial or complete linear membranous staining of viable tumor cells was considered positive PD-L1 staining and was included in the scoring.

Moreover, any membranous and/or cytoplasmic staining of mononuclear inflammatory cells within tumor nests and/or adjacent supporting stroma was considered positive PD-L1 staining and was included in the CPS numerator.

TMA cores without any viable tumor cells or those with <100 viable cells were considered unassessable. A total of 68 single cores were not evaluated due to <100 viable tumor cells.

**Fig. 1:**
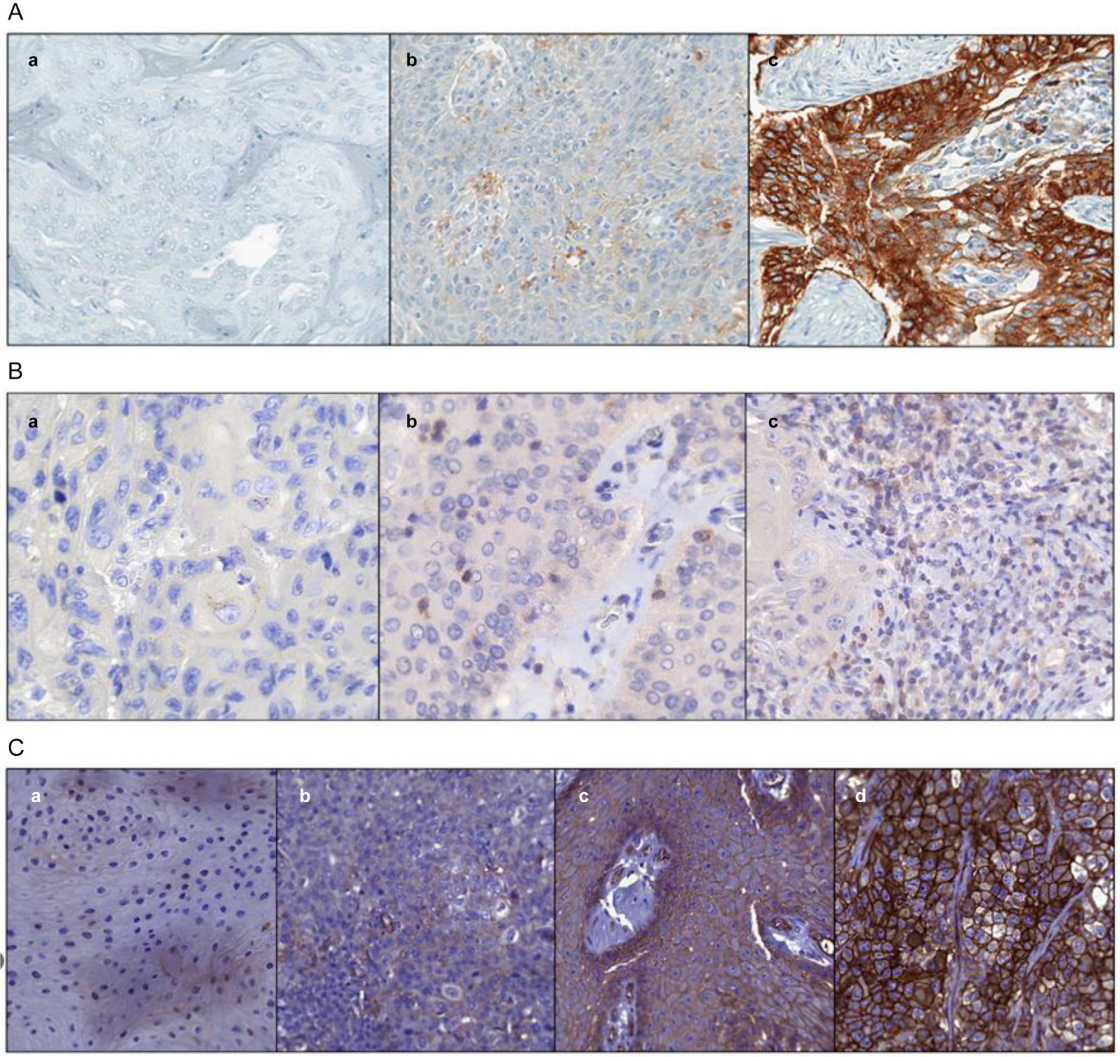
(A) PD-L1 expression in TMA penile SCC: representative images for (a) CPS < 1, (b) CPS ≥1, and (c) CPS ≥20 (original magnification 200×). (B) TIGIT expression in TMA penile SCC: representative images for (a) negative, (b) low, and (c) high TIGIT expression (original magnification 400×). (C) CD155 expression in TMA penile SCC: representative images for (a) negative, (b) low, (c) moderate, and (d) high CD155 expression (original magnification 200×). CD155 = cluster of differentiation 155; CPS = combined positive score; PD-L1 = programmed cell death ligand 1; SCC = squamous cell carcinoma; TIGIT = T-cell immunoglobulin and immunoreceptor tyrosine-based inhibitory motif domain; TMA = tissue microarray.

PD-L1 expression was assessed and divided into the following PD-L1 expression levels: CPS <1 = 0, CPS ≥1 = 1, and CPS ≥20 = 2 (Fig. 1A).

For statistical analyses, due to small numbers, patients were divided into two groups: patients with PD-L1 = 0 and patients with PD-L1 = 1–2.

#### 2.3.2 TIGIT

For each TMA core, TIGIT expression was assessed semiquantitatively by counting the number of TIGIT-positive lymphocytes manually in three randomly selected fields at high power (400×; Fig. 1B). TMA cores without viable tumor cells or those with <100 viable cells were excluded from evaluation.

For statistical analyses, patients were divided into two groups: TIGIT = 0–5 and TIGIT >5, with the cutoff based on the mean of positive cells, comparing patients having expression on or below the mean with patients having higher expression.

#### 2.3.3 CD155

CD155 expression was evaluated using a semiquantitative method based on the staining intensity in tumor cells. The intensity was scored as follows: 0 (no staining), 1 (low staining), 2 (moderate staining), and 3 (high staining). The staining intensity of CD155-positive cells was determined for all cells in each TMA core (Fig. 1C). TMA cores without viable tumor cells or those with <100 viable cells were excluded from evaluation.

For statistical analyses, due to small numbers, patients were divided into two groups: CD155 = 0–2 and CD155 = 3.

### 2.4 HPV detection

Tissue blocks were cut in sections (4 µm), and DNA was extracted using the QIAamp DNA FFPE Tissue Kit (Qiagen GmbH, Hilden, Germany). The polymerase chain reaction (PCR) method Anyplex II HPV28 was utilized for HPV DNA detection and genotyping (Seegene, Seoul, Korea), which detects 28 different genotypes together with the human gene beta-globin (*HBB).* Samples were run on a CFX96TM real-time PCR system (Bio-Rad Laboratories, Hercules, CA, USA).

### 2.5 Statistical analysis

The Kaplan-Meier method was used to estimate peCSS. Follow-up began at the date of diagnosis and continued until death, emigration, or the end of follow-up (December 31, 2020), whichever occurred first. The event of interest was death due to penile cancer, with deaths from other causes treated as censored at the time of death. Two-year peCSS estimates and corresponding 95% confidence intervals (CIs) were derived from the Kaplan-Meier survival curves. The median follow-up time was calculated using the reverse Kaplan-Meier method. Differences in survival between groups were assessed using the log-rank test.

Owing to the small study population and low percentage of missing data, multiple imputation was not considered appropriate for handling patients with missing data. A complete case analysis was performed.

R version 4.1.2 (R Foundation for Statistical Analysis, Vienna, Austria) was used for statistical analyses, *p* < 0.05 was considered statistically significant, and all tests were two sided.

## 3 Results

### 3.1 Follow-up, surgery, and perioperative oncological treatment

Of those without an event, the median follow-up was 50 mo for the entire group of 52 patients and 53 mo for the 33 patients who received oncological treatment.

Of the 52 patients, 47 underwent curative intent surgery to inguinal lymph nodes, 35 of whom also underwent curative intent surgery to pelvic lymph nodes. The five patients who did not undergo lymph node surgery had comorbidities that precluded surgery (four patients) or were considered inoperable at diagnosis (one patient). Of these five patients, one had chemotherapy but did not respond and received palliative radiotherapy later, one had palliative radiotherapy only, and the remaining three were considered too frail to receive any treatment.

Thirty-three patients received perioperative chemotherapy and/or radiotherapy in accordance with guidelines (Table 1). Perioperative in this context means either neoadjuvant (before any surgery, before any lymph node surgery, or before surgery of pelvic lymph nodes) or adjuvant (after all planned surgeries). Both neoadjuvant and adjuvant chemotherapy was used, while radiotherapy was adjuvant in all cases. Owing to the small sample size, all patients who received any perioperative oncological therapy were merged into one group to be compared with the group that did not receive any perioperative treatment. Of the 33 patients, 32 also underwent lymph node surgery as part of curative treatment.

**Table 1:** Description of patients; T stage; tumor grade; N stage; expression of PD-L1, TIGIT, and CD155; HPV status; perioperative oncological treatment; and surgery to lymph nodes

	Patients with indication who received perioperative oncological therapy	Patients with indication who did not receive perioperative oncological therapy		All patients with indication for perioperative oncological therapy
Number of patients	33	19		52
Age (yr) at diagnosis, median (interquartile range)	67 (49–74)	74 (71.5–77)		70 (58–75)
T stage, *n* (%)				
pT1a	5 (15)	–		5 (10)
pT1b	3 (9)	–		3 (6)
pT2	13 (39)	9 (47)		22 (42)
pT3	11 (33)	9 (47)		20 (38)
No value	1 (3)	1 (5)		2 (4)
Tumor grade, *n* (%)				
1	1 (3)	–		1 (2)
2	14 (42)	5 (26)		19 (37)
3	18 (55)	13 (68)		31 (60)
No value	–	1 (5)		1 (2)
N stage, *n* (%)				
pN1 + cN1	2 (6)	5 (26)		7 (13)
pN2 + cN2	4 (12)	2 (11)		6 (12)
pN3 + cN3	27 (82)	12 (63)		39 (75)
PD-L1 expression, *n* (%)				
0	9 (27)	4 (21)		13 (25)
1	12 (36)	8 (42)		20 (38)
2	12 (36)	7 (36)		19 (37)
TIGIT expression, *n* (%)				
0–5	22 (67)	14 (74)		36 (69)
>5	10 (30)	5 (26)		15 (29)
No value	1 (3)	–		1 (2)
CD155 expression, *n* (%)				
0	–	1 (5)		1 (2)
1	3 (9)	2 (11)		5 (10)
2	8 (24)	5 (26)		13 (25)
3	18 (55)	11 (58)		29 (56)
No value	4 (12)	–		4 (8)
HPV status, *n* (%)				
Positive	13 (39)	11 (58)		24 (46)
Negative	19 (58)	8 (42)		27 (52)
No value	1 (3)	–		1 (2)
Perioperative oncological treatment, *n* (%)				
Neoadjuvant chemotherapy + adjuvant radiotherapy	6 (18)			6 (12)
Neoadjuvant chemotherapy	15 (45)			15 (29)
Adjuvant radiotherapy	7 (21)			7 (13)
Adjuvant chemotherapy	5 (15)			5 (10)
Surgery to lymph nodes, *n* (%)				
Radical inguinal lymph node dissection	32 (97)	15 (79)		47 (90)
Pelvic lymph node dissection	28 (85)	7 (37)		35 (66)

CD155 = cluster of differentiation 155; HPV = human papillomavirus; PD-L1 = programmed cell death ligand 1; TIGIT = T-cell immunoglobulin and immunoreceptor tyrosine-based inhibitory motif domain.

Twenty-six patients received chemotherapy (21 neoadjuvant and five adjuvant), five of whom also received radiotherapy, while seven patients received radiotherapy alone. The reasons why 19 men did not receive treatment included the following: comorbidity or high age in ten cases, purportedly lack of indication for treatment in six cases, and rapid deterioration in three cases.

Six of the 21 patients treated in the neoadjuvant situation had this treatment before the study tissue sample was taken; the remaining 15 patients had treatment after the sample was taken.

In total, 27 of the 52 patients died of penile cancer during follow-up, 15 of 33 in the group that received perioperative oncological treatment and 12 of 19 in the group that did not receive treatment.

### 3.2 PD-L1, TIGIT, and CD155 expression and HPV status for the entire cohort

PD-L1, TIGIT, and CD155 were expressed widely in our cohort. Thirty-nine of 52 (75%) patients had PD-L1–positive tumors, 41 of 51 (80%) patients had TIGIT-positive tumors, and 47 of 48 (98%) patients had CD155-positive tumors. For a detailed description of the expression within the cohort, see Table 1. Twenty-four of 51 (47%) patients had HPV-positive cancer.

Data regarding PD-L1 expression were available for all 52 patients. Owing to insufficient material, data on TIGIT expression were missing for one patient, data on CD155 were missing for four patients, and data on HPV status were missing for one patient.

### 3.3 Survival analyses including penile cancer patients with indication for perioperative oncological treatment

**Fig. 2:**
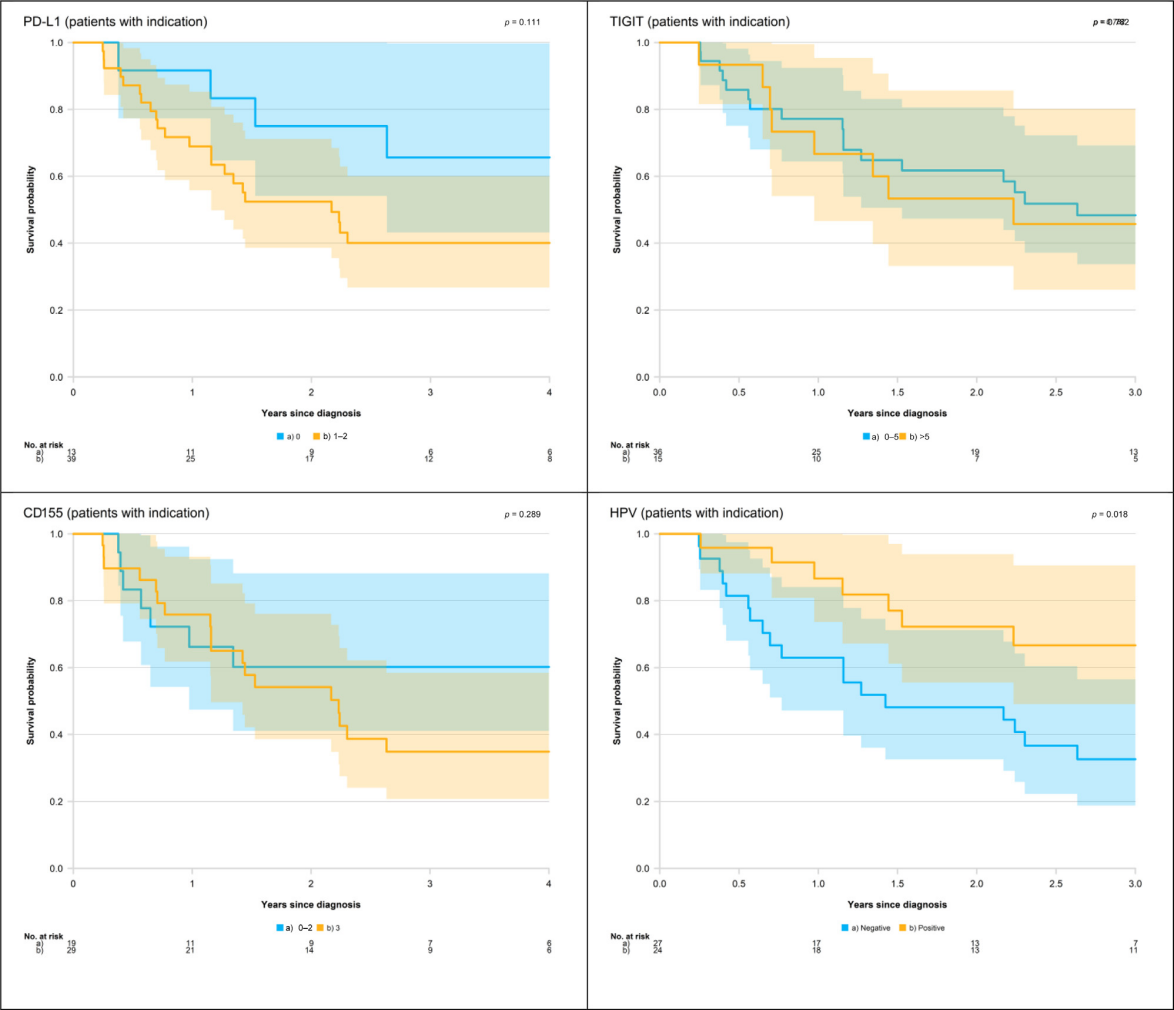
Kaplan-Meier curves for penile cancer–specific survival among patients who had indication for perioperative oncological treatment, by expression of PD-L1, TIGIT, CD155, and HPV. CD155 = cluster of differentiation 155; HPV = human papillomavirus; PD-L1 = programmed cell death ligand 1; TIGIT = T-cell immunoglobulin and immunoreceptor tyrosine-based inhibitory motif domain.

Initially, we performed Kaplan-Meier analyses to compare the survival probability of patients with high and low expression of the different biomarkers. Analyses were conducted separately for each biomarker, including all patients with indication for perioperative oncological treatment (Fig. 2).

The 2-yr peCSS rate for men with PD-L1 = 0 was 75% (95% CI: 54–100%) compared with 52% (39–71%) for patients with PD-L1 = 1–2. In men with TIGIT = 0–5, the 2-yr peCSS rate was 62% (95% CI: 47–80%), while for patients with TIGIT >5, it was 53% (33–86%). In patients with CD155 = 0–2, the peCSS rate at 2 yr was 60% (95% CI: 41–88%), while for patients with CD155 = 3, it was 54% (39–76%).

The differences in survival, found when comparing patients having low expression of PD-L1, TIGIT, or CD155 with those having high expression, were not statistically significant.

The 2-yr peCSS rate in patients with HPV+ tumors was 72% (95% CI: 56–94%) compared with 48% (33–71%) for patients with HPV– tumors. Patients with HPV+ tumors had statistically significantly better survival than those with HPV– tumors (*p* = 0.02).

### 3.4 Survival analyses including penile cancer patients receiving perioperative oncological treatment

**Fig. 3:**
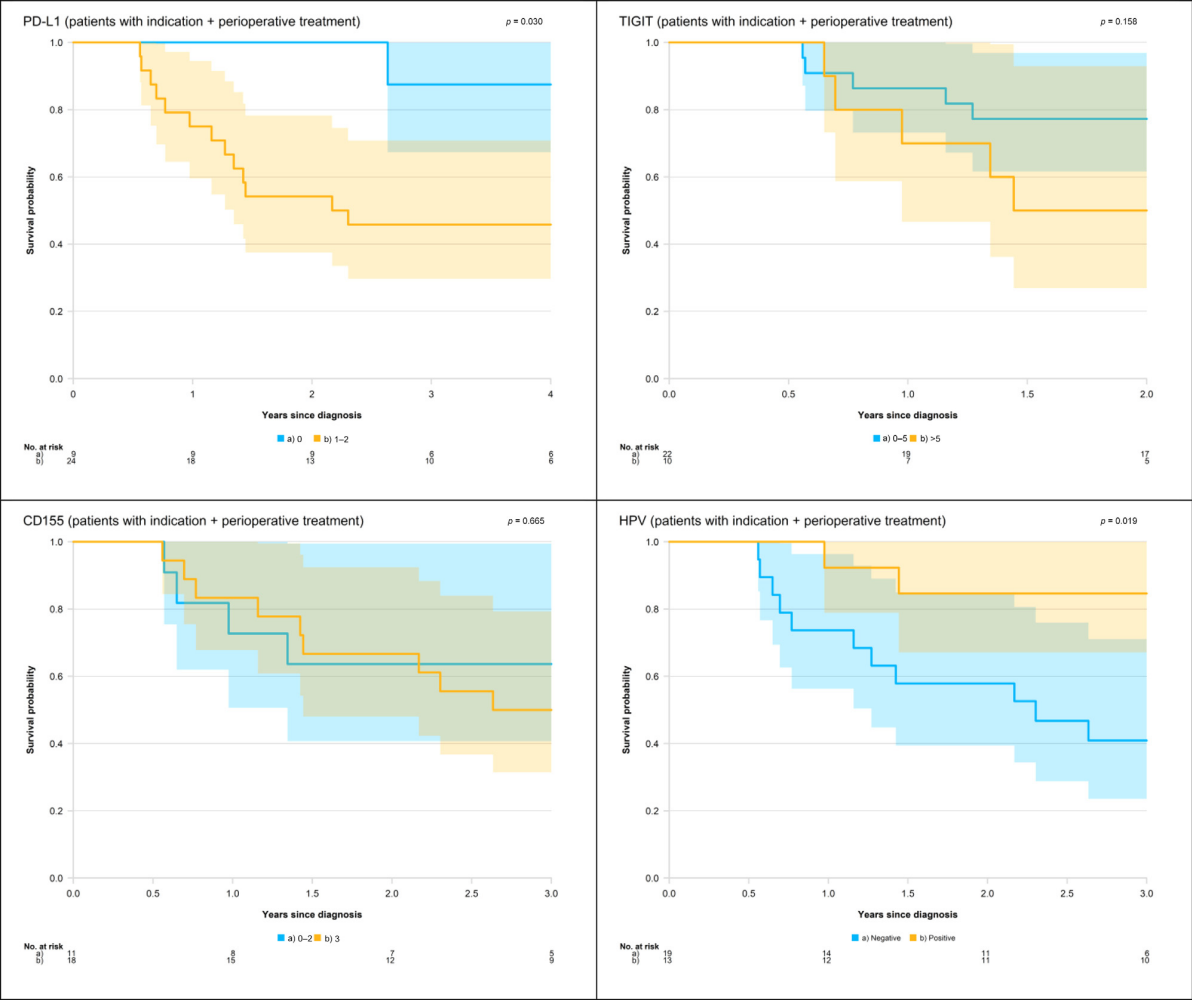
Kaplan-Meier curves for penile cancer–specific survival among patients who had indication for and received perioperative oncological treatment, by expression of PD-L1, TIGIT, CD155, and HPV. CD155 = cluster of differentiation 155; HPV = human papillomavirus; PD-L1 = programmed cell death ligand 1; TIGIT = T-cell immunoglobulin and immunoreceptor tyrosine-based inhibitory motif domain.

In the next step, we performed Kaplan-Meier analyses including only those patients who received oncological treatment (Fig. 3).

The 2-yr peCSS rate was 100% (95% CI 100–100%) in men with PD-L1 = 0 and 54% (37–78%) in men with PD-L1 = 1–2. Patients without PD-L1 expression had statistically significantly better survival than those with PD-L1 = 1–2 (*p* = 0.03).

For patients with TIGIT = 0–5, the peCSS rate at 2 yr was 77% (95% CI: 62–97%) compared with 50% (27–93%) in men with TIGIT >5.

In men with CD155 = 0–2, the 2-yr peCSS rate was 64% (95% CI: 41–99%), and in patients with CD155 = 3, it was 67% (48–92%).

No statistically significant difference in survival was found for TIGIT or CD155.

The peCSS rate at 2 yr was 85% (95% CI: 67–100%) in patients with HPV+ tumors compared with 58% (39–85%) in patients with HPV– tumors. Men with HPV+ tumors had statistically significantly better survival than those with HPV– tumors (*p* = 0.02).

## 4 Discussion

It is well established that an immunosuppressed tumor microenvironment (TME) promotes cancer progression, and the expression of various inhibitory immune checkpoint proteins have been proposed as prognostic markers in several malignancies. The most studied immune checkpoints are PD-1/PD-L1 and CTLA-4, although their value as predictive biomarkers for treatment response remains inconclusive in many malignancies and also in penile cancer. In the present study, we have evaluated the expression and prognostic value of PD-L1, as well as two additional immune checkpoint proteins, TIGIT and CD155, along with HPV status, in a cohort of men with lymph node metastasized penile cancer. The results indicate an immunosuppressed TME in the primary tumor, as evidenced by high expression of all three inhibitory checkpoint proteins.

Upregulation of PD-L1 expression is a mechanism by which tumors evade host immune responses. In the context of penile cancer, previous studies have reported PD-L1 positivity rates ranging from 32% to 67%. In the present study, 75% of patients exhibited PD-L1–positive tumors, which may be attributable to the advanced stage of the tumors in our cohort.

We found improved survival in penile cancer patients without PD-L1 expression, reaching statistical significance only for the group of patients who received oncological treatment, somewhat but not fully supporting the evidence that PD-L1 positivity is associated with poor prognosis in penile cancer [8,20].

We also observed frequent expression of TIGIT and CD155, both of which have been studied in other malignancies [[21], [22], [23]]. To our knowledge, this is the first evaluation of these biomarkers in penile cancer. TIGIT expression has been associated with poor prognosis in non–small cell lung cancer, particularly in combination with PD-L1 expression [24], as well as in muscle-invasive bladder cancer, where it serves as a potential predictive biomarker of an inferior response to chemotherapy [22]. These previous findings in other malignancies suggest that TIGIT, as part of the immunosuppressive TME, promotes tumor growth and could therefore be a prognostic and predictive marker, as well as a potential therapeutic target. We found high expression of TIGIT and CD155 in this cohort, but did not find evidence of prognostic value in penile cancer patients.

Consistent with previous studies, we found lower cancer-specific mortality in patients with HPV-related penile cancer [1,25]. This suggests that distinct mechanisms contribute to the pathogenesis of HPV-related and non–HPV-related penile cancer.

The limitations of this study must be acknowledged. The most significant of these include the small number of patients, retrospective design, and use of TMAs rather than whole tissue sections. Studies including few patients are an ever-present challenge in research on rare diseases, and larger studies are warranted to further assess the prognostic and predictive values of these biomarkers. Nevertheless, despite the limited cohort size and the use of TMAs, we were able to provide insights into an immunosuppressive TME and support the role of HPV status as a prognostic biomarker.

Treatment with chemotherapy and radiotherapy can induce a more immunosuppressive TME by increased expression of immunosuppressive proteins [[26], [27], [28]]. In our study, six of 21 patients had chemotherapy before the study tissue sample was taken, which could have influenced the expression of checkpoint proteins; since subgroups included few patients, no analyses were made on differences between groups based on treatment before or after tissue sampling, which is another limitation.

Future studies are needed to evaluate the TME in penile cancer with respect to immunosuppression and investigate the genetic differences between HPV-related and non–HPV-related penile cancer in greater detail.

Subcellular localization of PD-L1, TIGIT, and CD155 was not investigated in this study, but could provide valuable insights into molecular interactions and roles in immune evasion within the TME. This can be achieved in future studies through double immunohistochemical staining targeting a combination of the present evaluated markers along with markers specific to macrophages, T cells, or tumor cells. There are also several other checkpoint molecules that could prove to be important as prognostic and predictive biomarkers in penile cancer, such as CTLA-4, TIM3, and LAG3, molecules that were not included in this study but would be of great interest to investigate.

Future aims should be to explore the TME in penile cancer to identify novel prognostic and predictive biomarkers as well as possible therapeutic targets.

## 5 Conclusions

The high expression rates of all three immune checkpoint–related proteins observed in this study provide evidence of an immunosuppressive TME in advanced penile cancer. In addition, better prognosis was observed for patients with HPV-positive tumors. Our results indicate that penile cancer patients with advanced disease could benefit from therapies targeting the immune response to cancer. Future studies identifying, mapping, and targeting mechanisms employed by cancer cells to evade immune elimination, as well as trials of combining treatments targeting the immune response are needed.

  ***Author contributions*:** Emma Ulvskog had full access to all the data in the study and takes responsibility for the integrity of the data and the accuracy of the data analysis.

  *Study concept and design*: Ulvskog, Davidsson, Kirrander.

*Acquisition of data*: Ulvskog, Davidsson, Dorofte, Lillsunde-Larsson, Franceschini, Fiorentino.

*Analysis and interpretation of data*: Ulvskog, Davidsson, Persson, Kirrander, Dorofte, Lillsunde-Larsson, Franceschini, Fiorentino.

*Drafting of the manuscript*: Ulvskog, Davidsson.

*Critical revision of the manuscript for important intellectual content*: Kirrander, Persson, Dorofte, Lillsunde-Larsson, Franceschini, Fiorentino.

*Statistical analysis*: Persson.

*Obtaining funding*: Ulvskog.

*Administrative, technical, or material support*: None.

*Supervision*: Davidsson, Kirrander.

*Other*: None.

  ***Financial disclosures:*** Emma Ulvskog certifies that all conflicts of interest, including specific financial interests and relationships and affiliations relevant to the subject matter or materials discussed in the manuscript (eg, employment/affiliation, grants or funding, consultancies, honoraria, stock ownership or options, expert testimony, royalties, or patents filed, received, or pending), are the following: None.

  ***Funding/Support and role of the sponsor*:** Funding for this work has been obtained through the Agreement concerning research and education of doctors (ALF) from Region Örebro County and from the Research Committee of Region Örebro County, and the Steering Group for the National Penile cancer Register of Sweden. The funders played no role in the design of the study or in any part of the collection, analysis and interpretation of data.

  ***Acknowledgments*:** The project was made possible by the Swedish Penile Cancer Register. The Regional Cancer Centre of Mid-Sweden contributed with statistical analyses. Preliminary results on HPV status have been presented as a poster at the EUROGIN International Multidisciplinary HPV Congress 2024, poster number: P 30-1. Title: Oncological treatment to men with penile cancer, survival in correlation to treatment and HPV-status.
